# Long-Term Potentiation Enhances Neuronal Differentiation in the Chronic Hypoperfusion Model of Rats

**DOI:** 10.3389/fnagi.2018.00029

**Published:** 2018-02-15

**Authors:** Hayato Takeuchi, Masahiro Kameda, Takao Yasuhara, Tatsuya Sasaki, Atsuhiko Toyoshima, Jun Morimoto, Kyohei Kin, Mihoko Okazaki, Michiari Umakoshi, Ittetsu Kin, Ken Kuwahara, Yosuke Tomita, Isao Date

**Affiliations:** Department of Neurological Surgery, Okayama University Graduate School of Medicine, Dentistry and Pharmaceutical Sciences, Okayama, Japan

**Keywords:** LTP, neurogenesis, chronic hypoperfusion, hippocampus, mild cognitive impairment (MCI)

## Abstract

Several reports have shown that long-term potentiation (LTP) *per se* effectively enhances neurogenesis in the hippocampus of intact animals. If LTP can enhance neurogenesis in chronic hypoperfusion, this approach could potentially become a new therapeutic strategy for the restoration of cognitive function and for prevention from deterioration of mild cognitive impairment (MCI). Using an *in vivo* LTP model of rats, we examined whether LTP *per se* can enhance neurogenesis in hypoperfusion rats that underwent permanent bilateral common carotid artery occlusion (permanent 2-vessel occlusion, P2VO). High frequency stimulation (HFS) in the subacute phase after P2VO enhanced hippocampal cell proliferation and neurogenesis. However, most enhanced cell proliferation and neurogenesis was seen in the hypoperfusion rats that received HFS and for which LTP could finally be induced. In contrast, the same effect was not seen in the LTP induction in the chronic phase. The present findings, which reveal that most enhanced neurogenesis was seen in hypoperfusion rats for which LTP could be finally induced, could explain the ability of LTP-like activities such as learning paradigms and environmental stimuli to increase the rate of neurogenesis in the hippocampus even under hypoperfusion conditions. Moreover, the present findings, which reveal that LTP induction in the chronic phase after P2VO could not effectively enhance neurogenesis in the hypoperfusion rats, could indicate that patients with MCI and even middle-aged healthy control individuals should start LTP-like activities as early as possible and continue with these activities to prevent age-related deterioration of hippocampal function.

## Introduction

Cerebral hypoperfusion is generally associated with later cognitive decline (Kitagawa et al., 2009), and silent brain infarcts were shown to be a risk factor for mild cognitive impairment (MCI) in the Cardiovascular Health Study (Lopez et al., 2003). MCI, in turn, increases the risk of later progression to Alzheimer’s disease (Hirao et al., 2005) and non-Alzheimer dementia (Staekenborg et al., 2009). Initial reduced regional cerebral blood flow (rCBF) in patients with MCI is a useful marker for the prediction of rapid conversion to Alzheimer’s disease (Hirao et al., 2005). Severe white matter hyperintensities and a higher prevalence of lacunas in the basal ganglia on baseline magnetic resonance imaging in patients with MCI are useful markers for progression to non-Alzheimer dementia (Staekenborg et al., 2009). Taken together, cerebral hypoperfusion and silent infarction are an etiological factor for Alzheimer’s disease and one of the causes of non-Alzheimer dementia (e.g., vascular dementia). Patients with MCI experience impairment in activities of daily living (ADL) due to memory disturbance, yet they can maintain their physical health. MCI and its progression to Alzheimer’s disease or non-Alzheimer dementia is a problem not only for the individual but also for society. Unfortunately, methods for restoring cognitive function and for preventing the progression in patients with MCI are poorly understood, and this is an urgent issue in an aging society.

Previous reports have shown the decline of neurogenesis in the dentate gyrus (DG) during adulthood and aging (Seki and Arai, 1995; Kempermann et al., 2002). This decline raises the possibility that reduced neurogenesis may compromise hippocampal function and account, at least in part, for impaired learning and memory and cognitive deterioration in the elderly (Lazarov and Hollands, 2016). This implies that enhancing neurogenesis in adults and the elderly offers the possibility that patients with MCI could restore cognitive function and prevent progression to Alzheimer’s disease or non-Alzheimer dementia.

Because MCI is a preclinical condition of Alzheimer’s disease or non-Alzheimer dementia, a less invasive method for enhancing neurogenesis would be valuable for patients with this condition. In order to enhance neurogenesis, two types of approaches are available: the use of exogenous stem cells and the activation of endogenous stem cells. Regarding the use of exogenous stem cells, the goal is to replace the damaged area with transplanted exogenous stem cells, neural stem cells (Kameda et al., 2007), mesenchymal stem cells (Wang et al., 2010; Toyoshima et al., 2015), induced pluripotent stem cells (iPS cells; Zhang et al., 2011; Zwi-Dantsis et al., 2013), and others. However, using exogenous stem cells is likely to be more invasive than the activation of endogenous stem cells. Many researchers have examined a variety of approaches in order to enhance endogenous neurogenesis. To date, environmental enrichment (Seo et al., 2013) and rehabilitation (Ding et al., 2004) have been shown to enhance recovery from ischemic insults through the activation of endogenous stem cells in the subventricular zone (SVZ) and DG. Electrical stimulation has also shown the effectiveness of the activation of endogenous stem cells after ischemia (Baba et al., 2009; Morimoto et al., 2011). Deep brain stimulation may have an impact on the progression of symptoms in patients with Alzheimer’s disease (Laxton et al., 2010), and it has been shown to promote subgranular zone proliferation and facilitate spatial memory performance in mice (Stone et al., 2011). However, which type of electrical stimulation in these particular conditions (e.g., ischemia and Alzheimer’s disease) induces neurogenesis more effectively has not been sufficiently clarified.

Latent precursor cells in the hippocampus can be activated by potassium *in vitro* (Walker et al., 2008), and we previously showed that long-term potentiation (LTP) *per se* enhanced neurogenesis in the hippocampus of intact mice *in vivo* (Kameda et al., 2012). The perforant pathway (PP), mossy fibers and Schaffer collaterals are major afferent hippocampal pathways (Xiong et al., 2017), and they exhibit different biochemical and electrophysiological properties (Panja and Bramham, 2014; Wiera and Mozrzymas, 2015). LTP is the enhancement of synaptic transmission, and high-frequency stimulation (HFS) on the PP is one approach for inducing LTP. LTP has now been found in other brain structures (Horibe et al., 2014; Skiteva et al., 2017) and can be induced by other types of stimulation paradigms besides HFS (Sugimura et al., 2017). LTP is considered one of the mechanisms of memory (Tsien et al., 1996) and learning (Bliss and Collingridge, 1993). Whitlock et al. (2006) showed that learning actually induces LTP in CA1. Potassium and LTP are related to depolarization, which is the key for activation of latent hippocampal neurons (Walker et al., 2008; Kameda et al., 2012). If LTP can enhance neurogenesis after silent infarction or in chronic hypoperfusion, this approach could potentially become a new therapeutic strategy for the restoration of cognitive function and the prevention of progression in MCI patients. Consistent with this, using an *in vivo* LTP model of rats, we examined in this study whether LTP *per se* can enhance neurogenesis after permanent 2-vessel occlusion (P2VO), which has been established as a procedure to investigate the effects of chronic cerebral hypoperfusion on cognitive dysfunction and neurodegenerative processes (Farkas et al., 2007; Bang et al., 2013).

## Materials and Methods

### Ethics Statement

The use of animals in this study was approved by the Animal Care and Use Committee of Okayama University Graduate School of Medicine, Dentistry and Pharmaceutical Sciences (Okayama, Japan). All procedures were carried out in accordance with institutional guidelines, and all efforts were made to minimize suffering.

### Animals

Adult male Wistar rats (CLEA JAPAN, Inc., Tokyo, Japan; *n* = 33) weighing 200–250 g and aged 6 weeks at the beginning of the experiment were used in this study. They were housed in a temperature- and humidity-controlled room, which was maintained on a 12-h light/dark cycle, with free access to food and water.

### Hypoperfusion Model (Sancesario et al., 1991; Nanri et al., 1998; Busch et al., 2003; Farkas et al., 2007)

A hypoperfusion model was created through permanent bilateral common carotid artery occlusion (P2VO). All rats were deeply anesthetized with pentobarbital (35 mg/kg, i.p.), and the bilateral carotid arteries were carefully separated from the cervical sympathetic and vagal nerves through a ventral cervical incision. The bilateral common carotid arteries were then doubly ligated by 5–0 silk. Body temperature was maintained at around 37°C until the rats were completely awake after anesthesia (Katsumata et al., 2010). In the sham group, rats underwent the same surgical operation without ligation.

### Electrophysiology

For electrophysiological recording, the rats were anesthetized with pentobarbital (35 mg/kg, i.p., with supplemental injections as required) and placed in a stereotaxic frame, and body temperature was maintained at 37°C. Based on procedures from previous reports (Jay et al., 1999; Aldridge et al., 2005; Chun et al., 2006), rats were stereotaxically implanted with two epoxy-insulated microelectrodes (thickness 100 μm, UJ-70-02-1.0; FHC Inc., Bowdoinham, ME, USA), a stimulating electrode in the PP (location: ipsilaterally 4.5 mm lateral to lambda), and a recording electrode in the hilus of the DG (location: 3.7 mm posterior to Bregma and 2.5 mm lateral to the midline). Both electrodes were lowered slowly to the point where maximal field excitatory postsynaptic potential (fEPSP) was evoked by low-frequency test stimuli. Although increased electrode penetration would result in better electrode positioning for electrophysiological recording, it would induce brain tissue trauma, resulting in trauma-induced neurogenesis. In order to prevent this from occurring, electrode positioning was limited to four penetrations. After generating an input/output curve, a stable 30-min baseline was recorded (stimulus pulse width 50 μs at 0.05 Hz). After recording the baseline, tetanus (400 Hz, 10 trains of six pulses, five times) was delivered to induce LTP. The post-HFS baseline was then recorded for 60 min (stimulus pulse width 50 μs at 0.05 Hz). The stimulus intensity was kept constant throughout, including during tetanus. All recorded signals were amplified by DAM50 (World Precision Instruments, Sarasota, FL, USA), filtered, digitized by Digidata1440 (Molecular Devices, San Jose, CA, USA), and analyzed offline using pClamp 10 (Molecular Devices, San Jose, CA, USA).

Rats were then assigned to one of two groups: the LTP (+) group, if fEPSP response was >120% and population (POP) spike was >200% of the baseline at 60 min after HFS and the LTP (−) group, in which rats received HFS but LTP was not induced (that is, the fEPSP response and POP spike did not fulfill the criteria above).

### Experimental Design

#### LTP Induction in Hypoperfusion Model in the Subacute Phase

**Figure 1:**
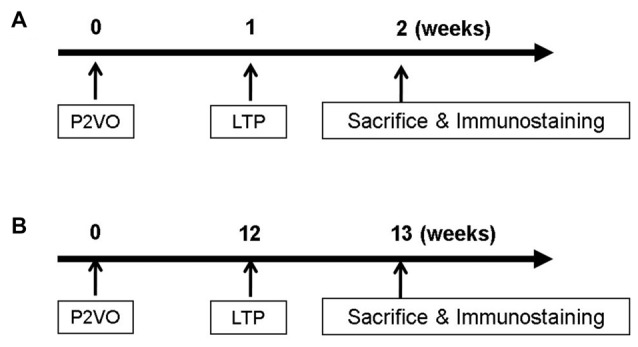
In order to evaluate neurogenesis by long-term potentiation (LTP) induction in the hypoperfusion model, high frequency stimulation (HFS) was delivered into the hypoperfusion rats in the subacute phase (1 week after permanent 2-vessel occlusion, P2VO surgery; **A**) or in the chronic phase (12 weeks after P2VO surgery; **B**).

In order to evaluate neurogenesis by LTP induction in the hypoperfusion model, rats (weighing 200–250 g) underwent P2VO surgery and received HFS 1 week postoperatively (Figure 1A). Before LTP induction, rats weighing under 200 g were excluded because their condition was not stable enough to undergo the electrophysiological procedure due to severe ischemic brain after P2VO surgery. Finally, rats were classified into four groups: the sham group, which underwent 2VO surgery but without ligation and without insertion of electrodes; the P2VO group, which underwent P2VO surgery and had electrodes positioned but no stimulation; the LTP(−) group, which underwent P2VO surgery but for which LTP was not induced by HFS; and the LTP(+) group, which underwent P2VO surgery and for which LTP was induced by HFS.

#### LTP Induction in Hypoperfusion Model in the Chronic Phase

In order to evaluate neurogenesis by LTP induction in the hypoperfusion model (Kawaguchi et al., 2002) in the chronic phase, rats underwent P2VO surgery and received HFS 12 weeks after P2VO surgery (Figure 1B). Rats were again classified into four groups: sham, P2VO, LTP(−) and LTP(+).

### Immunohistochemistry

All rats were euthanized with an overdose of pentobarbital (100 mg/kg) at 1 week after LTP induction and perfused transcardially with 200 ml of cold phosphate buffered saline (PBS) and 200 ml of 4% paraformaldehyde (PFA) in PBS. Brains were removed and post-fixed in the same fixative overnight at 4°C, and subsequently stored in 30% sucrose in PBS until completely submerged. The brains were coronally sectioned at a thickness of 40 μm.

The following primary antibodies (final dilution and source) were used for tissue staining: rabbit anti-Ki67 (1:100; Novus Biologicals, Littleton, CO, USA) and mouse anti-DCX (1:100; Novus Biologicals) to evaluate proliferation and differentiation. Before immunohistochemical analysis, sections were washed three times for 5 min in PBS. Sections were incubated overnight at 4°C in primary antibody (diluted in PBS containing 10% normal horse serum and 0.3% Triton-X), washed with PBS, and then incubated with regular secondary antibodies conjugated to Cy3 and FITC and DAPI (1:1000 Molecular Probes, Eugene, OR, USA) for 1 h at room temperature. Sections were then washed three times in PBS for 5 min each and coverslipped with Gelmount (Biomedia Corp., Foster City, CA, USA).

### Image Analyses

BZX-700 (Keyence, Osaka, Japan) was used to acquire images. In the DG, the numbers of Ki67-positive and Ki67/DCX double-positive cells along the entire *z*-axis (40 μm) were counted every 12 sections throughout the hippocampus. False double-positive cells caused by overlay signals from different cells were excluded by rotating in orthogonal planes. The total numbers of Ki-67 positive and Ki67/DCX double-positive cells were then calculated.

### Statistical Analysis

LTP was expressed as the percentage change from the baseline of fEPSP and POP spike. The number of Ki67 and Ki67/DCX double-positive cells obtained from each rat was calculated. Mean values are presented with standard deviations (SDs).

All data were analyzed using statistical software JMP4.0.5J. A two-tailed *t*-test was used for comparisons between two groups. Comparisons among more than two groups were performed using repeated measures of analysis of variance (ANOVA) with a *post hoc* Tukey-Kramer honestly significant difference (HSD) test. Statistical significance was defined as *p* < 0.05.

## Results

We examined whether LTP can stimulate latent hippocampal precursor cells in the chronic hypoperfusion. HFS was applied to the PP *in vivo* to induce LTP (LTP[+] group). In some animals, this protocol failed to induce LTP, allowing for a no-LTP control (LTP[−] group) to be examined.

### Changes in Body Weight

**Figure 2:**
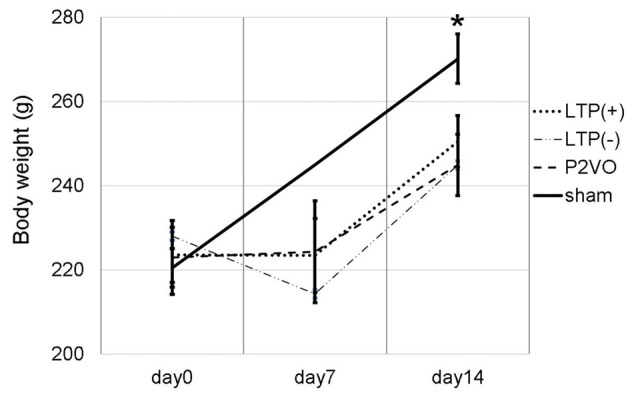
We measured the body weight at day 0 (before P2VO surgery), at day 7 (1 week after P2VO surgery but before LTP induction), and at day 14 (1 week after LTP induction) in each group. There were no statistically significant differences in the body weight among the LTP(+), the LTP(−) and the P2VO groups throughout the experimental period. There were, however, statistically significant differences between these three groups and the sham group (*N* = 6, 6, 6, 3, LTP(+), LTP(−), P2VO, sham, respectively). *p < 0.05.

We measured the body weight of rats to evaluate the invasiveness of P2VO surgery and electrophysiological surgery. The body weight at day 0 (before P2VO surgery), at day 7 (1 week after P2VO surgery but before LTP induction), and at day 14 (1 week after LTP induction) in each group is shown in Figure 2. There was no statistically significant differences among the body weight in the LTP(+), the LTP(−) and the P2VO groups throughout the experimental period, although there were statistically significant differences between these three groups and the sham group (*N* = 6, 6, 6, 3, LTP(+), LTP(−), P2VO, sham, respectively). The body weight in all groups, except for the sham group, did not increase between day 0 and day 7, but it did increase between day 7 and day 14. This change in body weight indicates that the condition of rats after P2VO surgery in these three groups can be regarded as similar and that the hypoperfusion effect on the hippocampus can also be regarded as similar. Electrophysiological surgery (either tetanus stimulation for the LTP [+] group and the LTP [−] group or electrode insertion without stimulation for the P2VO group) seems to have little impact in terms of an invasive effect on body weight.

Moreover, in the experiment of LTP induction in the chronic phase, the body weight temporally decreased after P2VO surgery in the acute phase. The body weight subsequently increased until electrophysiological surgery 12 weeks after P2VO surgery (each group *N* = 3, 440 ± 45.8, 430 ± 50, 450 ± 5.8 and 480 ± 30, for LTP(+), LTP(−), P2VO and sham, respectively), although one rat did unfortunately die in the acute phase.

### Electrophysiological Recording

**Figure 3:**
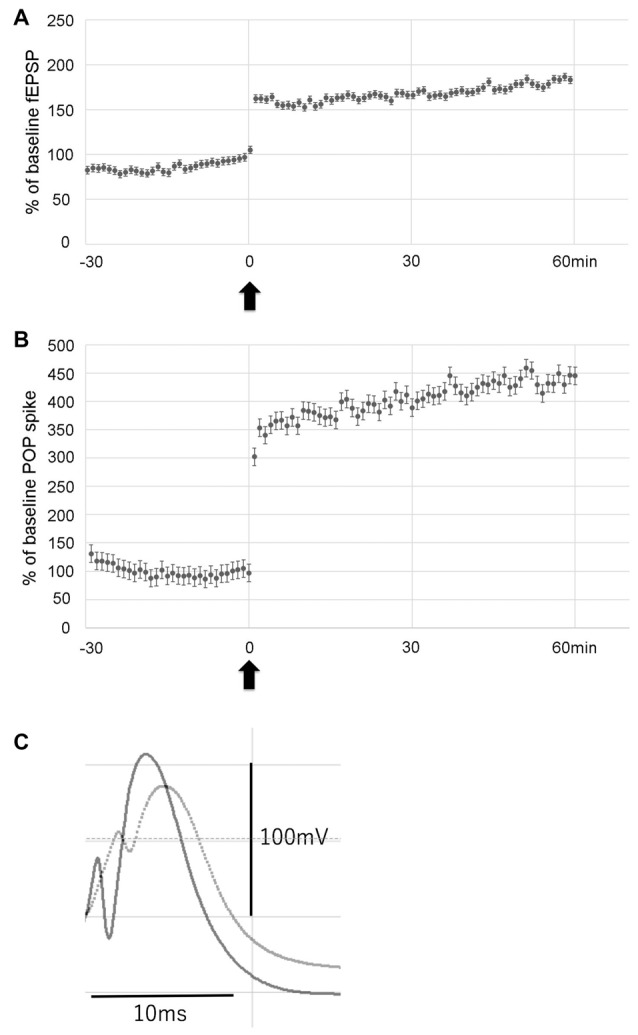
Data showing the mean field excitatory postsynaptic potential (fEPSP) slope (± standard deviation, SD) and the mean POP spike (± SD) as a percentage of baseline. Rats in the LTP(+) group (*N* = 6) showed stable potentiation throughout post-HFS baseline recording, as measured by both fEPSP slope **(A)** and POP spike **(B)**. Arrow indicates delivery of HFS. Error bars indicate SD. Evoked potentials recorded before (dotted line) and after LTP (solid line) are shown **(C)**.

To examine the effect of LTP induction on hippocampal neurogenesis in the hypoperfusion model, LTP was induced by HFS on the PP. Throughout post-HFS baseline recording for 60 min, rats in the LTP(+) group (*N* = 6) showed stable enhanced fEPSP (>150% of baseline) and POP spike (>300% of baseline; Figures 3A–C).

### Immunohistochemistry

**Figure 4:**
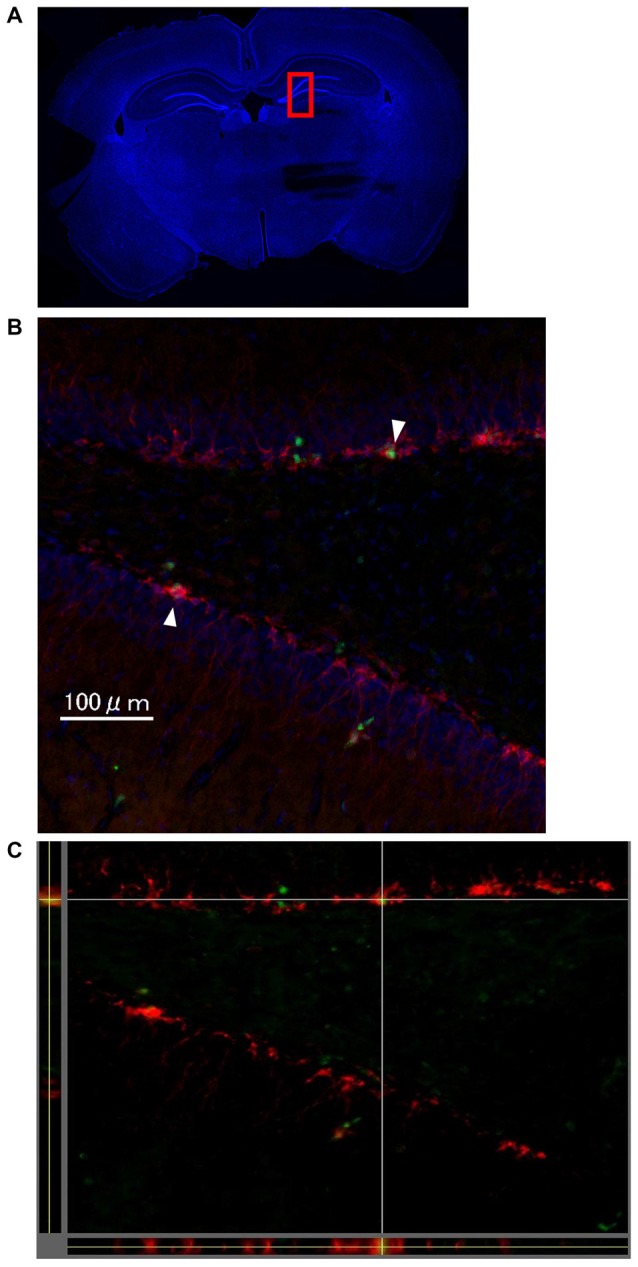
The images show representative immunohistochemical staining in dentate gyrus (DG; Bregma −4.0 mm) on the stimulated side in the LTP(+) group. There were some Ki67 (green)/DCX (red) double-positive cells in the DG (arrowhead; **A**: very low magnification with DAPI staining, **B**: high magnification of rectangle area (red) of **A,C**: orthogonal view of **B**, green: Ki67, red: DCX, blue: DAPI).

One week after LTP induction, each group of rats was sacrificed, and immunohistochemical analysis using Ki-67 and DCX was performed to evaluate neurogenesis. As seen in Figures 4A–C, there were some Ki67/DCX double-positive cells (arrowheads), which indicates that proliferated cells differentiated into immature neurons.

#### Evaluation of Neurogenesis

##### Cell proliferation

**Figure 5:**
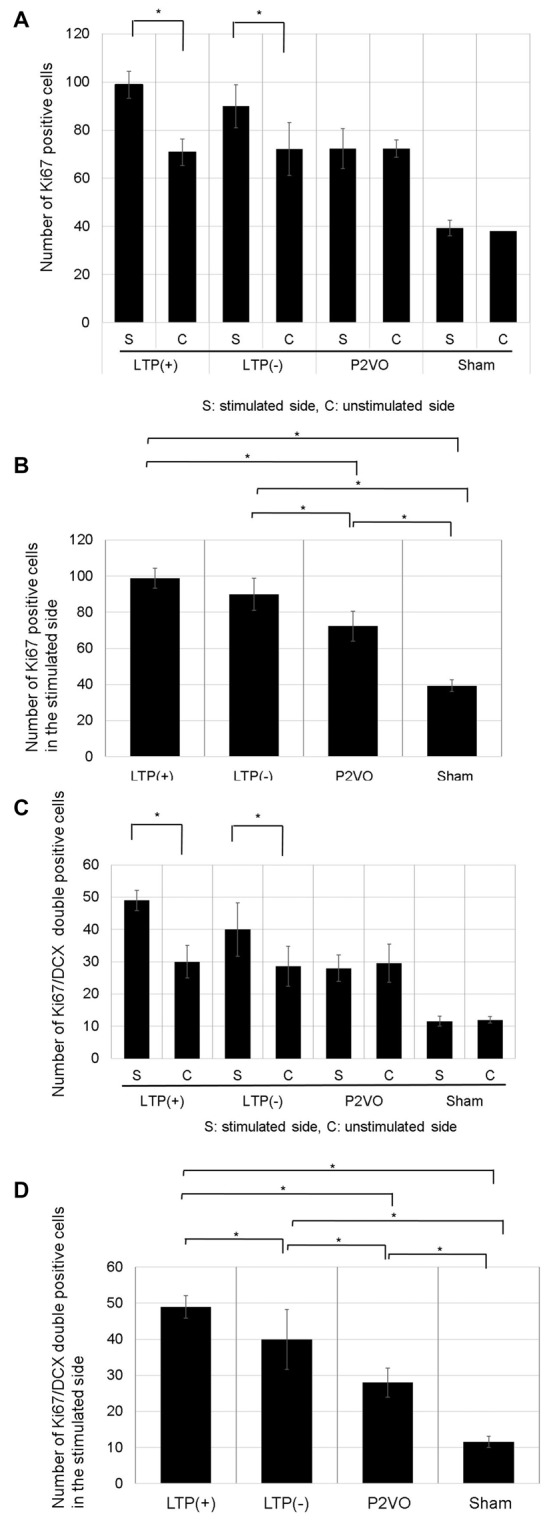
The effect of LTP induction on precursor cell proliferation and differentiation was assessed by the number of Ki67-positive cells and Ki67/DCX double-positive cells. **(A)** The number of Ki67-positive cells in the DG was significantly enhanced in the stimulated hippocampi of the LTP(+) and LTP(−) groups, relative to the unstimulated hemisphere (*N* = 6, 6, 6, 3, LTP(+), LTP(−), P2VO, sham, respectively). **(B)** In the stimulated hippocampi, the hypoperfusion effect in the P2VO group induced enhanced proliferation, compared with the sham group. Although HFS in the LTP(+) and LTP(−) groups induced enhanced proliferation in the stimulated hippocampi compared with the P2VO group, the greatest number of Ki67-positive cells was found in the LTP(+) group, compared with the other three groups (*N* = 6, 6, 6, 3, LTP(+), LTP(−), P2VO, sham, respectively). However, there was no statistical difference between the LTP (+) and LTP(−) groups in the Tukey-Kramer honestly significant difference (HSD) test. **(C)** Following HFS, the number of Ki67/DCX double-positive cells in the DG was significantly enhanced in the stimulated hippocampus of the LTP(+) and LTP(−) groups, compared with the unstimulated hippocampus (*N* = 6, 6, 6, 3, LTP(+), LTP(−), P2VO, sham, respectively). **(D)** In the stimulated hippocampi, the hypoperfusion effect in the P2VO group induced enhanced neurogenesis, compared with the sham group. Although HFS in the LTP(+) and LTP(−) groups induced enhanced neurogenesis in the stimulated hippocampi compared with the P2VO group, there was the statistically greatest number of Ki67/DCX double-positive cells in the LTP(+) group, compared with the other groups (*N* = 6, 6, 6, 3, LTP(+), LTP(−), P2VO, sham, respectively). *p < 0.05.

There was no significant difference in the number of Ki-67 positive cells between stimulated and unstimulated hippocampi in the P2VO and sham group. In contrast, the number of Ki-67 positive cells was significantly enhanced in the stimulated hippocampi in the LTP(+) and the LTP(−) groups, relative to the unstimulated hemisphere, which indicates that HFS induced proliferation (Figure 5A, *p* < 0.05, *N* = 6, 6, 6, 3, LTP(+), LTP(−), P2VO, sham, respectively). In the stimulated hippocampi, the number of Ki-67 positive cells was significantly enhanced in the P2VO group compared to the sham group, which might reflect on the hypoperfusion effect in the acute phase. Although HFS in the LTP(+) and LTP(−) groups induced enhanced proliferation in the stimulated hippocampi compared with the P2VO group, there was the greatest number of Ki67-positive cells in the LTP(+) group, compared with the LTP(−), the P2VO, and the sham groups. However, there was no statistically significant difference between the LTP(+) group and the LTP(−) group in the Tukey-Kramer HSD test. This means that HFS increased proliferation, and the most enhanced proliferation was observed in the rats that received HFS and for which LTP could finally be induced; however, there was no statistically significant difference between LTP(+) and LTP(−), which implies that some latent hippocampal progenitors had already been activated by the hypoperfusion effect (Figure 5B, *F*_(3,17)_ = 48.8905, *p* < 0.0001, *N* = 6, 6, 6, 3, LTP(+), LTP(−), P2VO, sham, respectively).

##### Neuronal differentiation

There was no significant difference in the number of Ki-67/DCX double-positive cells between stimulated and unstimulated hippocampi in the P2VO and sham group. In contrast, the number of Ki-67/DCX double-positive cells was significantly enhanced in the stimulated hippocampi of the LTP(+) and the LTP(−) groups, relative to the unstimulated hemisphere, which indicates that HFS induced neuronal differentiation (Figure 5C, *p* < 0.05, *N* = 6, 6, 6, 3, LTP(+), LTP(−), P2VO, sham, respectively). In the stimulated hippocampi, the number of Ki-67/DCX double-positive cells was significantly enhanced in the P2VO group compared to the sham group, which might reflect on the hypoperfusion effect in the acute phase. Although HFS in the LTP(+) and LTP(−) groups induced enhanced neuronal differentiation in the stimulated hippocampi compared with the P2VO group, there was the greatest number of Ki67/DCX double-positive cells in the LTP(+) group among all groups. Moreover, there was a statistically significant difference between the LTP(+) and LTP(−) groups in the Tukey-Kramer HSD test. This indicates that, statistically, most enhanced neuronal differentiation was observed in the rats that received HFS and for which LTP could finally be induced, although HFS enhanced neuronal proliferation in the LTP(+) and LTP(−) groups without statistical difference (Figure 5D, *F*_(3,17)_ = 38.9391, *p* < 0.0001, *N* = 6, 6, 6, 3, LTP(+), LTP(−), P2VO, sham, respectively).

**Figure 6:**
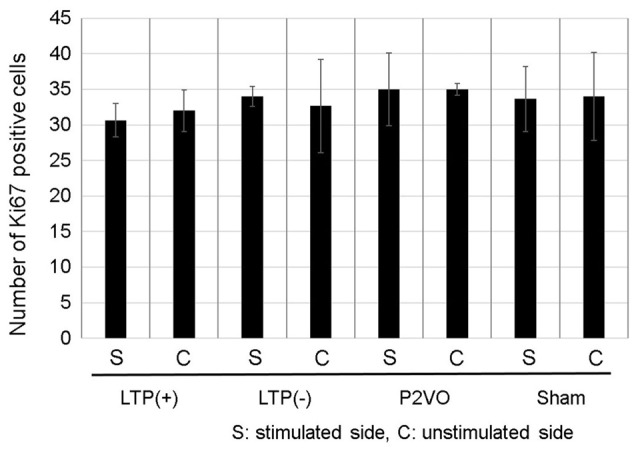
Compared with the sham group, the hypoperfusion effect could not enhance neurogenesis in the DG in the chronic phase following P2VO. Although LTP induction in the subacute phase after P2VO effectively enhanced neurogenesis in the DG, LTP induction in the chronic phase following P2VO could not enhance neurogenesis (each group *N* = 3).

To examine whether LTP induction in the chronic phase can induce neurogenesis in the chronic hypoperfusion model of rats, LTP was induced 12 weeks after P2VO surgery. There was no statistically significant difference, however, in the Ki-67 positive cells in the stimulated hippocampus among the four groups. Additionally, the number of Ki-67 positive cells in the stimulated hippocampi was not statistically different than that in the unstimulated hemisphere. Moreover, there were no Ki-67/DCX double-positive cells in any of the groups (Figure 6, each group *N* = 3).

## Discussion

In this study, we showed that HFS could induce cell proliferation in the hippocampus of hypoperfusion rats, yet statistically most enhanced neuronal differentiation was seen in hypoperfusion rats for which LTP was successfully induced. Nevertheless, LTP induction in the chronic phase could not induce enhanced neurogenesis.

Compared to the sham group, the body weight of rats that underwent P2VO decreased initially. Subsequently, the body weight recovered until the electrophysiology experiment up to 12 weeks after P2VO surgery. This indicates that rats with P2VO surgery are exposed to sufficient hypoperfusion insult and can be expected to survive for the long term. Compared to previous reports that used intact rats and mice, our aim in this study was to examine whether LTP can induce neurogenesis in the hypoperfusion model. In our preliminary experiment using mice, we could not generate a field potential by PP stimulation due to severe ischemic damage after 2VO (data not shown). Because rats have a developed posterior circulation, the severity of hippocampal damage after P2VO surgery in rats is usually milder than that in mice and gerbils (Qiu et al., 2016). Because of this developed posterior circulation, a P2VO model using rats is a more suitable chronic hypoperfusion model than that using mice or gerbils (Farkas et al., 2007). The extent of the development of posterior circulation differs, however, for each rat. Unfortunately, there is no feasible method for predicting the extent of the development of posterior circulation before the experiments. Based on this information, we excluded rats whose body weight was less than 200 g before LTP induction after P2VO because we anticipated the possibility that these rats (body weight <200 g before LTP induction after P2VO) could not generate a field potential due to severe ischemic insult. Rats that could fulfill these criteria (>200 g in the acute phase after P2VO surgery), however, could survive up to 12 weeks (until the LTP induction).

To visualize proliferative cells, we used Ki67 rather than Bromodeoxyuridine (BrdU). Ki67 marks proliferating cells during late G1, S, M and G2 phases of the cell cycle (Namba et al., 2005). On the other hand, BrdU is a thymidine analog that incorporates the DNA of dividing cells during the S-phase of the cell cycle (Taupin, 2007). The quantification of Ki67-positive cells has been shown to reflect cellular proliferation in a manner consistent with BrdU labeling in the adult DG (Eadie et al., 2005). Based on this information, we used Ki-67 for the evaluation of neurogenesis since it does not have any toxic influence when evaluating neurogenesis.

Previous reports showed LTP *per se* induce neurogenesis in the adult hippocampus rat (Bruel-Jungerman et al., 2006) and mice (Kameda et al., 2012) These reports provide the evidence for how synaptic activity associated with functions encoded by LTP, such as learning and memory, can specifically regulate neurogenesis at the precursor level in the adult hippocampus. Here we have demonstrated that HFS in the hypoperfusion hippocampus increased the number of proliferative Ki67 positive cells and neuronally committed Ki67/DCX double-positive cells *in vivo* as seen in the LTP(+) and LTP(−) groups, compared with the P2VO and sham groups. The approximately 40% and 30% increase (LTP[+] group and LTP[−] group, respectively) in precursor number relative to unstimulated controls obtained by the HFS indicates that the hippocampus in hypoperfusion rats contains a large pool of precursor cells that can be regulated by physiological inputs. Most importantly, statistically, most enhanced neuronal differentiation was seen in the LTP(+) group, providing the first evidence for how synaptic activity associated with functions encoded by LTP can specifically regulate neurogenesis at the precursor level in hypoperfusion rats. Our previous experiments using intact mice showed animals that received HFS, but failed to demonstrate that LTP (LTP[−] group) showed no change in hippocampal neurogenesis (Kameda et al., 2012). The hypoperfusion effect after P2VO surgery was responsible for this discrepancy. Although we have shown here that LTP leads to neuronal differentiation most effectively (with statistical significance) in hypoperfusion rats, the exact mechanism by which LTP activates precursor cells and enhances neurogenesis most effectively requires further investigation.

The present findings, which reveal that most enhanced cell proliferation and statistically most enhanced neuronal differentiation were seen in the LTP(+) group, could explain the ability of LTP-like activities such as learning paradigms and environmental stimuli to increase the rate of neurogenesis in the hippocampus even in hypoperfusion conditions. However, the present findings reveal that LTP induction in the chronic phase could not enhance neurogenesis in the chronic hypoperfusion rats. This result indicates there is a time-window to enhance neurogenesis by LTP induction after hypoperfusion insult. On the other hand, chronic hypoperfusion model rats do not appear severely ill because they can gain weight after P2VO surgery and electrophysiological surgery. From this point of view, we expected that the rats in the LTP(+) group could experience enhanced neurogenesis through LTP induction. However, neither the rats in the LTP(−) group nor the rats in the LTP(+) group showed enhanced neurogenesis through LTP induction in the chronic phase.

We used 6-week-old rats at the start of experiment and waited for 12 weeks after P2VO surgery before inducing LTP. Therefore, at the point of LTP induction, the rats were 4.5 months old. It is well known that neurogenesis is dependent on an animal’s age (Madroñal et al., 2010). Since the extent of neurogenesis gets progressively weaker with age, the extent of neurogenesis would decrease 12 weeks after P2VO surgery. Although endogenous neurogenesis in aged rats is much less extensive than in young adult rats, 4.5-month-old rats would not be considered aged. Taken together, gradual and continuous deterioration of degenerative damage might occur in chronic hypoperfusion rats after P2VO surgery (Farkas et al., 2007), which might, in turn, result in a weaker response by LTP induction in the chronic phase.

In this study, we failed to enhance neurogenesis through chronic phase LTP induction in the hypoperfusion model although we were able to succeed through acute phase LTP induction. The question remains as to how we can apply the results of this study to clinical situations or our daily lives while keeping in mind that enhancing neurogenesis is not without negative side effects. Cho et al. (2015) showed that ablation of adult neurogenesis before pilocarpine-induced acute seizures in mice lead to a reduction in chronic seizure frequency and ablation of neurogenesis normalized epilepsy-associated cognitive deficits. Enhancing neurogenesis is not always beneficial. In fact, enhancing neurogenesis in epilepsy is an especially poor approach unlike in other central nervous disease conditions (Rotheneichner et al., 2013). Of course, merely enhancing neurogenesis is not sufficient for restoring the damaged function. The type of disease (e.g., ischemia or epilepsy) and the severity of the disease would produce different results after enhancing neurogenesis. We regard our hypoperfusion model of rats as representative of patients with MCI. Under clinical conditions, Del Felice et al. (2014) reported that 4% of patients diagnosed with MCI showed improvement through anti-epileptic drugs, which implies epileptic amnestic syndrome misdiagnosed as MCI or dementia. This report, however, also implies that the MCI patients are essentially different from epilepsy patients with cognitive impairment. Moreover, Qiu and Folstein (2006) reported that 14%–44% of patients with MCI can exhibit reversion once they are diagnosed MCI. Taken together, patients with MCI are truly not healthy controls, but they have the potential to self-repair their damaged functions. Previouas reports showed that running exercise enhance not only neurogenesis but also LTP (Patten et al., 2013). Whitlock et al. (2006) showed that learning enhanced LTP. Running exercise and learning can therefore be regarded as LTP-related activities. Gruart et al. (2006) showed that LTP saturation resulted in learning and memory impairments. Fortunately, the LTP-saturation state does not last for a long time since a previous report showed LTP lasted nearly 1 year, yet the magnitude of LTP decreases daily (Abraham et al., 2002). On the other hand, it is difficult to detect the timing of LTP saturation in our daily lives. Hullinger and Burger (2015) reported that repeated training from an early age to old age in animals with learning impairment identified early in life could enhance cognitive function and that repeated training could ultimately prevent age-related cognitive decline. Taken together, early diagnosis and repeated training or LTP-like activity are essential for preventing age-related cognitive decline.

By conducting further research, we will hopefully be able to identify the mechanisms underlying the association between LTP and the modulation of precursor proliferation, differentiation, and survival, which would result in finding a more efficient method for enhancing endogenous neurogenesis. The discovery of such a method could more efficiently help restore cognitive function and prevent progression to Alzheimer’s disease or non-Alzheimer dementia in patients with MCI.

## Author Contributions

HT performed most of the research, with additional contributions from MK, TY, TS, AT, JM, KKin, MO, MU, IK, KKuwahara, YT and ID. HT and MK designed the project and wrote the manuscript with contributions from TY, JM, KKin and ID.

## Conflict of Interest Statement

The authors declare that the research was conducted in the absence of any commercial or financial relationships that could be construed as a potential conflict of interest.
